# Geometric least squares means ratios for the analysis of *Plasmodium falciparum *in vitro susceptibility to antimalarial drugs

**DOI:** 10.1186/1475-2875-6-156

**Published:** 2007-11-26

**Authors:** Michel Vaillant, Piero Olliaro

**Affiliations:** 1 Clinical Epidemiology and Public Health Unit, Centre for Health Studies, Centre de Recherche Publique (CRP)-Santé, Luxembourg; 2 Unité 3677, Bases thérapeutiques des inflammations et infections, Université Victor Segalen Bordeaux II, Bordeaux, France; 3 UNICEF/UNDP/WB/WHO Special Programme for Research & Training in Tropical Disease, Geneva, Switzerland

## Abstract

**Background:**

The susceptibility of microbes such as *Plasmodium falciparum *to drugs is measured in vitro as the concentration of the drug achieving 50% of maximum effect (IC_50_); values from a population are summarized as geometric means. For antimalarial drugs, as well as for antibiotics, assessing changes in microbe susceptibility over time under drug pressure would help inform treatment policy decisions, but no standard statistical method exists as yet.

**Methods:**

A mixed model was generated on log_e_-transformed IC_50 _values and calculated geometric least squares means (GLSM) with 90% confidence intervals (CIs). In order to compare IC_50_s between years, GLSM ratios (GLSMR) with 90%CIs were calculated and, when both limits of the 90%CIs were below or above 100%, the difference was considered statistically significant. Results were compared to those obtained from ANOVA and a generalized linear model (GLM).

**Results:**

GLSMRs were more conservative than ANOVA and resulted in lower levels of statistical significance. The GLSMRs approach allowed for random effect and adjustment for multiple comparisons. GLM was limited in the number of year-to-year comparisons by the need for a single reference year. The three analyses yielded generally consistent results.

**Conclusion:**

A robust analytical method can palliate inherent limitations of in vitro sensitivity testing. The random effects GLSMRs with adjustment for multiple comparisons and 90%CIs require only assumptions on the mixed model to be applied. Results are easy to display graphically and to interpret. The GLMSRs should be considered as an option for monitoring changes in drug susceptibility of *P. falciparum *malaria and other microbes.

## Background

*Plasmodium falciparum*, the species causing most of the malaria burden in the world, can be grown in culture, which makes it possible to measure parasite drug susceptibility *in vitro *[1]. Results are generally expressed as IC_50 _(the concentration achieving 50% of maximum effect). Turnidge *et al *[2] presented a new method to evaluate cut-off values to define antibacterial resistance. However, while methodologies are well established for antimicrobials [3], it is not possible, for the majority of drugs, to confidently classify a strain as resistant or sensitive because validated thresholds are not available, unlike the situation with antibiotics [4].

Of particular interest in *P. falciparum *drug susceptibility is not the sensitivity profile of an isolate from a given patient, but rather the monitoring of the sensitivity patterns to drugs when submitted to drug pressure in a given area [5]. This information may contribute to inform decision as to choice of drugs for the treatment of malaria. However, evaluating trends over time poses methodological problems, mainly because (i) the high variability of results makes conventional statistical methods, such as the analysis of variance (ANOVA), inadequate; (ii) the statistical unit is not the individual patient isolate but the composite population of parasites within an individual. Due to this distribution variability, these data are usually presented as geometric mean. A key issue in the analysis of these data is detecting trends over time. However, there is still no standard statistical approach for this [6], without loss of information.

Methods used in pharmacokinetic studies deal with similar problems in dealing with datasets with respect to drug disposition in an individual or a population of individuals [7-9]. Bioequivalence between drug formulations is typically assessed by using the analysis of variance of log_e_-transformed data from a cross-over design with the null hypothesis expressed by: H_0_: *μ*_T _= *μ*_R _where *μ*_T _and *μ*_R _represent the log-transformed expected bioavailability parameters of the test and reference formulations respectively [10,11]. A 90% confidence interval (CI) for the ratio test/reference for bioavailability parameters is constructed by using the equation: μT−μR±S2nt0.05(1),υ
 MathType@MTEF@5@5@+=feaafiart1ev1aaatCvAUfKttLearuWrP9MDH5MBPbIqV92AaeXatLxBI9gBaebbnrfifHhDYfgasaacPC6xNi=xH8viVGI8Gi=hEeeu0xXdbba9frFj0xb9qqpG0dXdb9aspeI8k8fiI+fsY=rqGqVepae9pg0db9vqaiVgFr0xfr=xfr=xc9adbaqaaeGacaGaaiaabeqaaeqabiWaaaGcbaacciGae8hVd02aaSbaaSqaaiabdsfaubqabaGccqGHsislcqWF8oqBdaWgaaWcbaGaemOuaifabeaakiabgglaXkabdofatnaakaaajuaGbaWaaSaaaeaacqaIYaGmaeaacqWGUbGBcqWG0baDdaWgaaqaaiabicdaWiabc6caUiabicdaWiabiwda1iabcIcaOiabigdaXiabcMcaPiabcYcaSiab=v8a1bqabaaaaaWcbeaaaaa@43B3@ where S is the square root of the mean square error from the analysis of variance, n is the number of subjects per period, t_0.05(1) _is the critical value of t at *α *= 0.05 with *υ *the degrees of freedom.

In the case of susceptibility testing conducted over time, when subjects are grouped by the year of sampling, the variance in the ANOVA of parasite susceptibility (expressed as IC_50_) can be separated out into the contributions of the parasite, the subject and the time of measurement. The ANOVA will allow for the year of measurement and test whether the variability between years occurs at random or not. The ratio of the mean sum of squares of the parameter (e.g. IC_50_) to the error mean sum of squares in the ANOVA will give an F-statistic to test the null hypothesis: H_0_: *μ*_year _= *μ*_year0_. This will provide a test of whether the arithmetic mean of IC_50_s measured from a given year is identical to the mean of IC_50_s obtained in the reference year.

An underlying assumption in order to use the ANOVA is the normality of the residuals (the difference between an individual value and the mean of the sample it belongs to, *x*_*i *_- x¯
 MathType@MTEF@5@5@+=feaafiart1ev1aaatCvAUfKttLearuWrP9MDH5MBPbIqV92AaeXatLxBI9gBaebbnrfifHhDYfgasaacPC6xNi=xH8viVGI8Gi=hEeeu0xXdbba9frFj0xb9qqpG0dXdb9aspeI8k8fiI+fsY=rqGqVepae9pg0db9vqaiVgFr0xfr=xfr=xc9adbaqaaeGacaGaaiaabeqaaeqabiWaaaGcbaGafmiEaGNbaebaaaa@2D64@.) However, verifying the null hypothesis of identity between means may not be possible with distributions of residuals that are generally neither normal nor log-normal. Furthermore, the sample size is most often too small with respect to the variance for the comparison of means. The high variability of data leads to large error variability in terms of error sum of squares. Thus, the detection of a difference will be difficult to interpret since it will be a function of the variability of data and sample size for each year. Mixed models can be used when there are different levels of clustering in the observations. One can assume that there is a grouping per year and a random part of measurements within years due to the subject and the parasites strains the subject is infected with. It allows the user to analyse samples (here: years) with unequal sample sizes and to relax the assumption of independently and identically distributed residuals while accounting for the data structure in a more flexible way [12].

For malaria, with the introduction of new treatment regimens such as the Artemisinin-containing Combination Therapies (ACTs) [13], it is important to evaluate whether, with parasites being exposed to drug pressure, the amounts of drug needed to inhibit parasite growth departs from that of a reference year, prior to and during deployment to monitor the evolution of drug susceptibility. In addition, appropriate statistical methods are needed to account for the variability in the determination of IC_50_s in order to properly inform treatment policy decisions.

Several approaches were explored to describe the trends over time of parasite in vitro drug susceptibility of the parasite using a dataset collected in Casamance (south-western Senegal) during 2000–2004 slightly adapted for the purpose from Agnamey et al [14].

## Methods

### Mixed model

A fixed effect model can be expressed as y_ij _= *μ *+ t_j _+ e_ij _where j is the year of measurement, y_ij _the observation on year j for patient I, *μ *the overall mean (also referred to as intercept in statistical softwares), t_j _the relative effect of year j, *μ *+ t_j _is the mean effect for the year j and e_ij _the residual variance for year j on the i^th ^patient[12]. The model allows for the patient effect, in which case the formula becomes y_ij _= *μ *+ p_i_+ t_j _+ e_ij _where p_i _represents the i^th ^patient effect. Instead of defining some effects as constants in the model, one could consider them as arising from independent samples with a normal distribution[12], i.e. as random effects. The model containing both fixed and random effects can then be referred to as a mixed model [12,15]. The underlying assumptions to using mixed models are: normally distributed residuals, normally distributed random effects and residuals independent of the random effects.

### GLSMRs calculations

A mixed linear model of log_e_-transformed values was estimated whereby the year was considered as fixed and the intercept as a random effect. The isolates were from different subjects each year.

From the mixed model, t statistics of standardized pair wise differences were calculated as y¯i−y¯jσ^ij
 MathType@MTEF@5@5@+=feaafiart1ev1aaatCvAUfKttLearuWrP9MDH5MBPbIqV92AaeXatLxBI9gBaebbnrfifHhDYfgasaacPC6xNi=xH8viVGI8Gi=hEeeu0xXdbba9frFj0xb9qqpG0dXdb9aspeI8k8fiI+fsY=rqGqVepae9pg0db9vqaiVgFr0xfr=xfr=xc9adbaqaaeGacaGaaiaabeqaaeqabiWaaaGcbaqcfa4aaSGaaeaacuWG5bqEgaqeamaaBaaabaGaemyAaKgabeaacqGHsislcuWG5bqEgaqeamaaBaaabaGaemOAaOgabeaaaeaaiiGacuWFdpWCgaqcamaaBaaabaGaemyAaKMaemOAaOgabeaaaaaaaa@3833@ where *i *and *j *are the indices of two years, y¯i
 MathType@MTEF@5@5@+=feaafiart1ev1aaatCvAUfKttLearuWrP9MDH5MBPbIqV92AaeXatLxBI9gBaebbnrfifHhDYfgasaacPC6xNi=xH8viVGI8Gi=hEeeu0xXdbba9frFj0xb9qqpG0dXdb9aspeI8k8fiI+fsY=rqGqVepae9pg0db9vqaiVgFr0xfr=xfr=xc9adbaqaaeGacaGaaiaabeqaaeqabiWaaaGcbaGafmyEaKNbaebadaWgaaWcbaGaemyAaKgabeaaaaa@2EED@ and y¯j
 MathType@MTEF@5@5@+=feaafiart1ev1aaatCvAUfKttLearuWrP9MDH5MBPbIqV92AaeXatLxBI9gBaebbnrfifHhDYfgasaacPC6xNi=xH8viVGI8Gi=hEeeu0xXdbba9frFj0xb9qqpG0dXdb9aspeI8k8fiI+fsY=rqGqVepae9pg0db9vqaiVgFr0xfr=xfr=xc9adbaqaaeGacaGaaiaabeqaaeqabiWaaaGcbaGafmyEaKNbaebadaWgaaWcbaGaemOAaOgabeaaaaa@2EEF@ are the least square means (LSM) for years *i *and *j *and σ^ij
 MathType@MTEF@5@5@+=feaafiart1ev1aaatCvAUfKttLearuWrP9MDH5MBPbIqV92AaeXatLxBI9gBaebbnrfifHhDYfgasaacPC6xNi=xH8viVGI8Gi=hEeeu0xXdbba9frFj0xb9qqpG0dXdb9aspeI8k8fiI+fsY=rqGqVepae9pg0db9vqaiVgFr0xfr=xfr=xc9adbaqaaeGacaGaaiaabeqaaeqabiWaaaGcbaacciGaf83WdmNbaKaadaWgaaWcbaGaemyAaKMaemOAaOgabeaaaaa@3091@ is the square-root of the estimated variance of y¯i−y¯j
 MathType@MTEF@5@5@+=feaafiart1ev1aaatCvAUfKttLearuWrP9MDH5MBPbIqV92AaeXatLxBI9gBaebbnrfifHhDYfgasaacPC6xNi=xH8viVGI8Gi=hEeeu0xXdbba9frFj0xb9qqpG0dXdb9aspeI8k8fiI+fsY=rqGqVepae9pg0db9vqaiVgFr0xfr=xfr=xc9adbaqaaeGacaGaaiaabeqaaeqabiWaaaGcbaGafmyEaKNbaebadaWgaaWcbaGaemyAaKgabeaakiabgkHiTiqbdMha5zaaraWaaSbaaSqaaiabdQgaQbqabaaaaa@3300@. In this model, LSMs are predicted population means from log_e_-transformed values. Consequently, assuming that Logeyiyj=Logeyi−Logeyj
 MathType@MTEF@5@5@+=feaafiart1ev1aaatCvAUfKttLearuWrP9MDH5MBPbIqV92AaeXatLxBI9gBaebbnrfifHhDYfgasaacPC6xNi=xH8viVGI8Gi=hEeeu0xXdbba9frFj0xb9qqpG0dXdb9aspeI8k8fiI+fsY=rqGqVepae9pg0db9vqaiVgFr0xfr=xfr=xc9adbaqaaeGacaGaaiaabeqaaeqabiWaaaGcbaGaemitaWKaem4Ba8Maem4zaC2aaSbaaSqaaiabdwgaLbqabaqcfa4aaSaaaeaacqWG5bqEdaWgaaqaaiabdMgaPbqabaaabaGaemyEaK3aaSbaaeaacqWGQbGAaeqaaaaakiabg2da9iabdYeamjabd+gaVjabdEgaNnaaBaaaleaacqWGLbqzaeqaaOGaemyEaK3aaSbaaSqaaiabdMgaPbqabaGccqGHsislcqWGmbatcqWGVbWBcqWGNbWzdaWgaaWcbaGaemyzaugabeaakiabdMha5naaBaaaleaacqWGQbGAaeqaaaaa@4A9C@ and that the geometric mean is the antilog of the mean of log_e_-transformed values, the geometric least squares means ratio (GLSMR) can be calculated with the antilog of the y¯i−y¯j
 MathType@MTEF@5@5@+=feaafiart1ev1aaatCvAUfKttLearuWrP9MDH5MBPbIqV92AaeXatLxBI9gBaebbnrfifHhDYfgasaacPC6xNi=xH8viVGI8Gi=hEeeu0xXdbba9frFj0xb9qqpG0dXdb9aspeI8k8fiI+fsY=rqGqVepae9pg0db9vqaiVgFr0xfr=xfr=xc9adbaqaaeGacaGaaiaabeqaaeqabiWaaaGcbaGafmyEaKNbaebadaWgaaWcbaGaemyAaKgabeaakiabgkHiTiqbdMha5zaaraWaaSbaaSqaaiabdQgaQbqabaaaaa@3300@ (LSMs differences) extracted from the model (where y_i _and y_j _can be expressed as linear combination *l*_*i*_'*b *and *l*_*j*_'*b *of the parameter estimates). From these linear combinations the parameter estimates that define the LSMs, σ^ij
 MathType@MTEF@5@5@+=feaafiart1ev1aaatCvAUfKttLearuWrP9MDH5MBPbIqV92AaeXatLxBI9gBaebbnrfifHhDYfgasaacPC6xNi=xH8viVGI8Gi=hEeeu0xXdbba9frFj0xb9qqpG0dXdb9aspeI8k8fiI+fsY=rqGqVepae9pg0db9vqaiVgFr0xfr=xfr=xc9adbaqaaeGacaGaaiaabeqaaeqabiWaaaGcbaacciGaf83WdmNbaKaadaWgaaWcbaGaemyAaKMaemOAaOgabeaaaaa@3091@, (i.e. the standard deviation of the LSMs difference), can be estimated by σ^ij2=s2l′i(X′X)−lj
 MathType@MTEF@5@5@+=feaafiart1ev1aaatCvAUfKttLearuWrP9MDH5MBPbIqV92AaeXatLxBI9gBaebbnrfifHhDYfgasaacPC6xNi=xH8viVGI8Gi=hEeeu0xXdbba9frFj0xb9qqpG0dXdb9aspeI8k8fiI+fsY=rqGqVepae9pg0db9vqaiVgFr0xfr=xfr=xc9adbaqaaeGacaGaaiaabeqaaeqabiWaaaGcbaacciGaf83WdmNbaKaadaqhaaWcbaGaemyAaKMaemOAaOgabaGaeGOmaidaaOGaeyypa0Jaem4Cam3aaWbaaSqabeaacqaIYaGmaaGccuWGSbaBgaqbamaaBaaaleaacqWGPbqAaeqaaOGaeiikaGIafmiwaGLbauaacqWGybawcqGGPaqkdaahaaWcbeqaaiabgkHiTaaakiabdYgaSnaaBaaaleaacqWGQbGAaeqaaaaa@4068@. The confidence interval can be derived as (y¯i−y¯j)±tυ^,α2σ^ij
 MathType@MTEF@5@5@+=feaafiart1ev1aaatCvAUfKttLearuWrP9MDH5MBPbIqV92AaeXatLxBI9gBaebbnrfifHhDYfgasaacPC6xNi=xH8viVGI8Gi=hEeeu0xXdbba9frFj0xb9qqpG0dXdb9aspeI8k8fiI+fsY=rqGqVepae9pg0db9vqaiVgFr0xfr=xfr=xc9adbaqaaeGacaGaaiaabeqaaeqabiWaaaGcbaGaeiikaGIafmyEaKNbaebadaWgaaWcbaGaemyAaKgabeaakiabgkHiTiqbdMha5zaaraWaaSbaaSqaaiabdQgaQbqabaGccqGGPaqkcqGHXcqScqWG0baDdaWgaaWcbaacciGaf8xXduNbaKaacqGGSaaljuaGdaWcaaqaaiab=f7aHbqaaiabikdaYaaaaSqabaGccuWFdpWCgaqcamaaBaaaleaacqWGPbqAcqWGQbGAaeqaaaaa@42F6@.

GLSM ratios (GLSMRs) were calculated for each between-year comparison. An adjustment for multiple comparisons was done in order to control for the overall type 1 error rate using the Tukey-Kramer method (chosen because it allows for unequal sample size between years). GLSMRs were considered statistically different if both bounds of the CIs fell on either side of the value of 1 (or 100% in percentage values). Previously [14], we had used GLSMRs calculated without this adjustment and evaluated the 95%CIs.

### Standard statistical methods

Standard methods such as the one-way ANOVA were also used with the year as fixed factor to analyse the variations of IC_50_s over time. For non-normally distributed data (significant Kolmogorov-Smirnov test), a log_e_-transformation was applied. The Levene test for homogeneity of the variance was used and, in case of non-homogeneity, a Welch adjustment for the ANOVA. A non-parametric Kruskall-Wallis sign rank test was used when parametric analyses were not suitable. Pair-wise mean comparisons between years for each treatment were carried out following ANOVA with a Tukey adjustment. Normality of residuals was checked with a non significant Shapiro-Wilk test and normal probability plots. Concurrently a generalized linear model (GLM) was also estimated with the year as fixed factor using a normal probability function and an identity link function parameterization [16].

A p-value of < 0.05 was considered statistically significant. All tests were two-tailed. Statistical analyses were carried out with the statistical package SAS^® ^System version 9.1.3 (SAS Institute, Cary, NC, USA).

### Dataset

The dataset is an update of the one described in Agnamey *et al *[14]. Briefly, *in vitro *susceptibility of local isolates to chloroquine (CQ), quinine (QN), artemisinin (ART) and the amodiaquine metabolite monodesethylamodiaquine (MdAQ) were monitored the using the DELI test [17] before (1997) and during the deployment (2000–2004) of artesunate+amodiaquine combination in Mlomp, a village in the district of Oussouye in Casamance, Southern Senegal, where malaria is mesoendemic (25 infective bites/person-year) and transmission occurs year-round with a peak during the rainy season (July–December). Samples for the *in vitro *assay were from consecutive patients recruited as part of an observational study [18] with a *P. falciparum *mono-infection and parasitaemia ≥ 0.2% [19].

## Results

**Figure 1:**
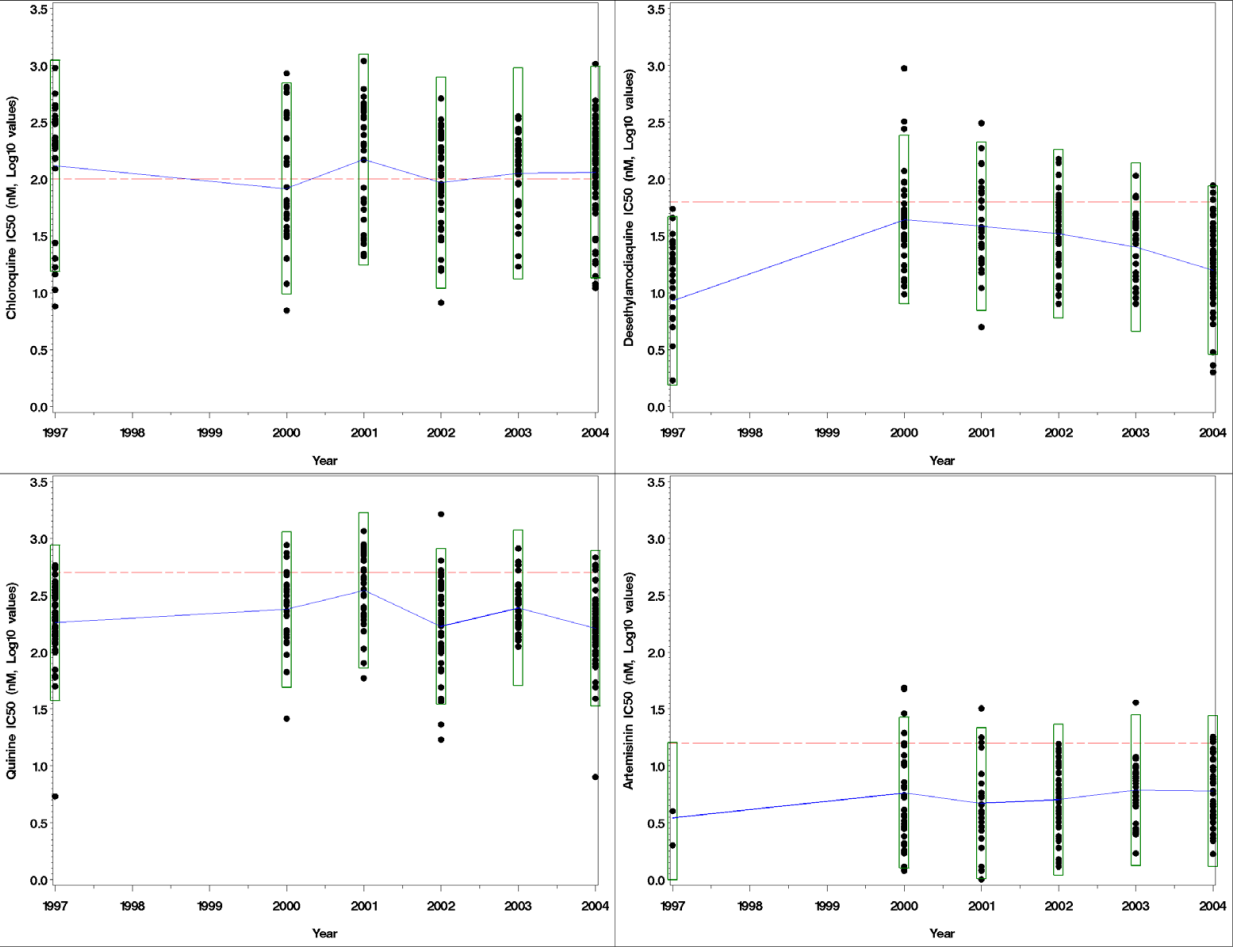
Scatterplots of the log_e_-transformed values of the quinine (QN), chloroquine (CQ), monodesethylamodiaquine (MdAQ), and artemisinin (ART) IC_50_s between years. The straight line is the cut-off value for susceptibility to the products (here: 100 nM for CQ, 60 nM for AQ, 500 nM for QN and 15 nM for ART [4]). The means of each year are connected with a trend line. The box around each year distribution of values represent two standard deviation of the mean.

IC_50_s from 242 subjects for CQ, 250 subjects for QN, 236 subjects for MdAQ and 183 subjects for ART were used in these statistical analyses [14]. The number of subjects with in vitro results was different among years and products tested (Table 1). Means with two standard deviations along with data distributions are plotted in Figure 1. Mean, Geometric means and GLSMs are presented together in Table 1. For all products except log-transformed ART, values were not normally distributed (Kolmogorov-Smirnov test p < 0.05).

**Table 1:** Means of raw and log_e_-transformed IC_50_s, geometric means and Geometric Least Squares Means.

1997				2000			2001			2002			2003			2004			Normality
						
Variable	N	Mean	STD	N	Mean	STD	N	Mean	STD	N	Mean	STD	N	Mean	STD	N	Mean	STD	p-value
	
Means (raw values)																			
CQ	31	236	203.4	33	173.2	223	31	251.4	236.7	46	131.3	104	34	143.8	91.03	67	171.5	157.2	0.01
QN	46	234.6	150.2	28	305.8	210.4	31	444	286.2	40	252.6	273.6	34	280.8	162.8	58	207.5	149.8	0.01
MdAQ	45	12.8	12.4	33	85.1	167.8	31	57.7	63.2	43	42.9	32.3	34	31.1	21.3	64	21.7	17.6	0.01
ART	10	3.6	0.8	31	9.8	12.0	31	6.1	6.6	40	5.7	4.5	34	7.4	5.9	36	7.5	5.0	0.01
Means (natural log values)																			
CQ	31	4.9	1.4	33	4.4	1.2	31	5	1.2	46	4.5	0.9	34	4.7	0.8	67	4.7	1	0.01
QN	46	5.2	0.8	28	5.5	0.8	31	5.9	0.7	40	5.1	0.9	34	5.5	0.5	58	5.1	0.8	0.04
MdAQ	45	2.1	0.9	33	3.8	1.0	31	3.6	0.9	43	3.5	0.7	34	3.2	0.6	64	2.8	0.9	0.04
ART	10	1.2	0.3	31	1.8	1.0	31	1.3	1	40	1.3	1.0	34	1.8	0.6	36	1.8	0.7	0.15
Geometric means																			
CQ	31	130.3	3.9	33	82.3	3.5	31	148.4	3.2	46	93.7	2.5	34	112.2	2.2	67	115.6	2.7	-
QN	46	181.3	2.3	28	237.5	2.2	31	350.7	2.1	40	169.0	2.6	34	244.7	1.7	58	162.4	2.1	-
MdAQ	45	8.5	2.5	33	43.8	2.7	31	38.5	2.4	43	33.1	2.1	34	25.3	1.9	64	15.8	2.4	-
ART	10	3.5	1.3	31	5.8	2.7	31	3.9	2.7	40	3.9	2.8	34	6.1	1.9	36	6.0	2.0	-
Geometric Least Squares Means																			
CQ	31	130.8	1.2	33	82.6	1.2	31	148.4	1.2	46	93.3	1.2	34	112.7	1.2	67	115	1.1	-
QN	46	181.3	1.1	28	238.2	1.2	31	350.1	1.1	40	168.4	1.1	34	245	1.1	58	162.2	1.1	-
MdAQ	45	8.5	1.1	33	44	1.2	31	38.5	1.2	43	33.1	1.1	34	25.3	1.2	64	15.9	1.1	-
ART	10	3.5	1.3	31	5.8	1.2	31	3.9	1.2	40	3.9	1.1	34	6.1	1.2	36	6.0	1.1	-

P-values are from the Kolmogorov-Smirnov test for normality. CQ = chloroquine; QN = quinine; MdAQ = monodesethylamodiaquine; ART = artemisinin

### Results of the analyses using the different methods

The ANOVA pair wise means comparisons (Table 2) showed no significant differences for CQ IC_50 _values. For MdAQ, statistically significant increases were observed between 1997 and 2000–2004, while there were decreases in IC_50_s between 2000–2004, 2001–2004 and 2002–2004. For QN, a statistically significant increase was observed between 1997 and 2001, and a decrease between 2001–2002 and 2001–2004 (Figure 1). For ART, no statistically significant differences were observed.

**Table 2:** Pairwise Least Squares Means comparisons following ANOVA of chloroquine (CQ), monodesethylamodiaquine (MdAQ), Quinine (QN) and Artemisinin (ART) log_e_-transformed IC_50 _(nM) between years.

	MdAQ				CQ				QN				ART			
year Comparison	Difference Between Means	Simultaneous 95% Confidence Limits		Sig.	Difference Between Means	Simultaneous 95% Confidence Limits		Sig.	Difference Between Means	Simultaneous 95% Confidence Limits		Sig.	Difference Between Means	Simultaneous 95% Confidence Limits		Sig.

1997 – 2000	-1.6	-2.3	-1.0	***	0.5	-0.4	1.4		-0.3	-0.9	0.4		-0.5	-1.6	0.5	
1997 – 2001	-1.5	-2.2	-0.8	***	-0.1	-1.0	0.8		-0.7	-1.3	0.0	***	-0.1	-1.2	0.9	
1997 – 2002	-1.4	-2.0	-0.7	***	0.3	-0.5	1.2		0.1	-0.5	0.6		-0.1	-1.1	0.9	
1997 – 2003	-1.1	-1.7	-0.4	***	0.1	-0.7	1.0		-0.3	-0.9	0.3		-0.6	-1.6	0.5	
1997 – 2004	-0.6	-1.2	-0.1	***	0.1	-0.6	0.9		0.1	-0.4	0.6		-0.5	-1.6	0.5	
2000 – 1997	1.6	1.0	2.3	***	-0.5	-1.4	0.4		0.3	-0.4	0.9		0.5	-0.5	1.6	
2000 – 2001	0.1	-0.6	0.8		-0.6	-1.5	0.3		-0.4	-1.1	0.3		0.4	-0.3	1.1	
2000 – 2002	0.3	-0.4	0.9		-0.1	-0.9	0.7		0.3	-0.3	1.0		0.4	-0.3	1.1	
2000 – 2003	0.6	-0.1	1.2		-0.3	-1.2	0.6		0.0	-0.7	0.6		-0.1	-0.8	0.7	
2000 – 2004	1.0	0.4	1.6	***	-0.3	-1.1	0.4		0.4	-0.2	1.0		0.0	-0.7	0.7	
2001 – 1997	1.5	0.8	2.2	***	0.1	-0.8	1.0		0.7	0.0	1.3	***	0.1	-0.9	1.2	
2001 – 2000	-0.1	-0.8	0.6		0.6	-0.3	1.5		0.4	-0.3	1.1		-0.4	-1.1	0.3	
2001 – 2002	0.2	-0.5	0.8		0.5	-0.4	1.3		0.7	0.1	1.4	***	0.0	-0.7	0.7	
2001 – 2003	0.4	-0.3	1.1		0.3	-0.6	1.2		0.4	-0.3	1.0		-0.5	-1.2	0.3	
2001 – 2004	0.9	0.3	1.5	***	0.3	-0.5	1.0		0.8	0.2	1.4	***	-0.4	-1.2	0.3	
2002 – 1997	1.4	0.7	2.0	***	-0.3	-1.2	0.5		-0.1	-0.6	0.5		0.1	-0.9	1.1	
2002 – 2000	-0.3	-0.9	0.4		0.1	-0.7	0.9		-0.3	-1.0	0.3		-0.4	-1.1	0.3	
2002 – 2001	-0.2	-0.8	0.5		-0.5	-1.3	0.4		-0.7	-1.4	-0.1	***	0.0	-0.7	0.7	
2002 – 2003	0.3	-0.4	0.9		-0.2	-1.0	0.6		-0.4	-1.0	0.2		-0.5	-1.1	0.2	
2002 – 2004	0.7	0.2	1.3	***	-0.2	-0.9	0.5		0.0	-0.5	0.6		-0.4	-1.1	0.2	
2003 – 1997	1.1	0.4	1.7	***	-0.1	-1.0	0.7		0.3	-0.3	0.9		0.6	-0.5	1.6	
2003 – 2000	-0.6	-1.2	0.1		0.3	-0.6	1.2		0.0	-0.6	0.7		0.1	-0.7	0.8	
2003 – 2001	-0.4	-1.1	0.3		-0.3	-1.2	0.6		-0.4	-1.0	0.3		0.5	-0.3	1.2	
2003 – 2002	-0.3	-0.9	0.4		0.2	-0.6	1.0		0.4	-0.2	1.0		0.5	-0.2	1.1	
2003 – 2004	0.5	-0.1	1.1		0.0	-0.8	0.7		0.4	-0.2	1.0		0.0	-0.7	0.7	
2004 – 1997	0.6	0.1	1.2	***	-0.1	-0.9	0.6		-0.1	-0.6	0.4		0.5	-0.5	1.6	
2004 – 2000	-1.0	-1.6	-0.4	***	0.3	-0.4	1.1		-0.4	-1.0	0.2		0.0	-0.7	0.7	
2004 – 2001	-0.9	-1.5	-0.3	***	-0.3	-1.0	0.5		-0.8	-1.4	-0.2	***	0.4	-0.3	1.2	
2004 – 2002	-0.7	-1.3	-0.2	***	0.2	-0.5	0.9		0.0	-0.6	0.5		0.4	-0.2	1.1	
2004 – 2003	-0.5	-1.1	0.1		0.0	-0.7	0.8		-0.4	-1.0	0.2		0.0	-0.7	0.7	

Using the GLM with 1997 as the reference year (Table 3), no relationship was found for CQ and ART, a significant positive estimate for MdAQ (IC_50_s decreased) and a significant positive estimate for QN in 2001, followed by a negative estimate (IC_50_s increased) in 2002.

**Table 3:** Generalized linear model (SAS System Proc Genmod) of chloroquine (CQ), monodesethylamodiaquine (MdAQ), quinine (QN) and artemisinin (ART) IC_50 _(nM) with the year as independent parameter (log_e_-transformed values and 1997 as the reference value).

		CQ			MdAQ			QN			ART		
Parameter		Estimate	Std Error	p	Estimate	Std Error	p	Estimate	Std Error	p	Estimate	Std Error	p

Intercept		4.9	0.2	<.0001	2.1	0.1	<.0001	5.2	0.1	<.0001	1.2	0.3	<.0001
year*	2000	-0.5	0.3	N.S.	1.6	0.2	<.0001	0.3	0.2	N.S.	0.5	0.3	N.S.
	2001	0.1	0.3	N.S.	1.5	0.2	<.0001	0.7	0.2	0.0003	0.1	0.3	N.S.
	2002	-0.3	0.2	N.S.	1.4	0.2	<.0001	-0.1	0.2	N.S.	0.1	0.3	N.S.
	2003	-0.1	0.3	N.S.	1.1	0.2	<.0001	0.3	0.2	N.S.	0.6	0.3	N.S.
	2004	-0.1	0.2	N.S.	0.6	0.2	0.0001	-0.1	0.2	N.S.	0.5	0.3	N.S.
Scale		1.1	0.0	-	0.8	0.0	-	0.8	0.0	-	0.8	0.0	-

**Figure 2:**
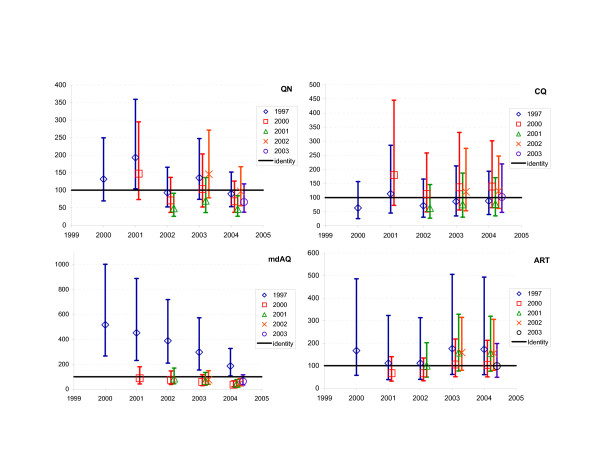
Geometric Least Squares Means Ratios of the IC_50_s to quinine (QN), chloroquine (CQ), monodesethylamodiaquine (MdAQ), and artemisinin (ART). *The legend indicates the reference year for the ratio. The x-axis indicates the year tested in the ratio. A logarithmic scale was applied for QN*.

GLSMR (Table 4) of CQ IC_50_s showed no significant differences, consistent with a stable response to CQ over the study period (Figure 2). For MdAQ, there were statistically significant increases between 1997 and 2000–2004. From 2000 to 2004, there was a decrease in IC_50_s which was significant between 2000 and 2004, 2001 and 2004, as well as 2002 and 2004. QN showed an increase between 1997 and 2001 and 2001–2002, and a significant decrease between 2002–2004. For ART, no statistically significant changes were found (Figure 2).

**Table 4:** IC_50_s for chloroquine (CQ) quinine (QN), monodesethylamodiaquine (MdAQ) and artemisinin (ART) in nM. Geometric Least Squares Means Ratio between years.

		MdAQ			CQ			QN			ART		
Variable	2 one-sided 90% limits	GLS Mean Ratio (%)	90%CI	2sided t p-value	GLS Mean Ratio (%)	90%CI	2sided t p-value	GLS Mean Ratio (%)	90%CI	2sided t-value	GLS Mean Ratio (%)	90%CI	2sided t p-value

1997 vs 2000	[80,125]	516.7	[266.4,1002.3]	<0.0001	63.2	[25.5,156.7]	N.S.	131.4	[69.3,249.2]	N.S.	166.8	[57.2,486.0]	N.S.
1997 vs 2001	[80,125]	452.3	[230.3,888.2]	<0.0001	113.5	[45.1,285.4]	N.S.	193.1	[103.8,359.2]	0.005	110.9	[38.0,323.0]	N.S.
1997 vs 2002	[80,125]	388.2	[209.6,719.2]	<0.0001	71.3	[30.7,165.8]	N.S.	92.9	[52.1,165.4]	N.S.	110.8	[39.2,313.4]	N.S.
1997 vs 2003	[80,125]	297.1	[154.0,573.0]	<0.0001	86.2	[35.0,212.3]	N.S.	135.1	[73.9,247.2]	N.S.	175.6	[61.0,505.7]	N.S.
1997 vs 2004	[80,125]	186.3	[106.1,326.9]	0.003	88.0	[40.0,193.5]	N.S.	89.5	[52.8,151.6]	N.S.	172.5	[60.3,493.5]	N.S.
2000 vs 2001	[80,125]	87.5	[42.5,180.4]	N.S.	179.6	[72.4,445.3]	N.S.	147.0	[73.3,294.9]	N.S.	66.5	[31.5,140.3]	N.S.
2000 vs 2002	[80,125]	75.1	[38.5,146.7]	N.S.	112.9	[49.3,258.3]	N.S.	70.7	[36.6,136.5]	N.S.	66.4	[32.9,134.2]	N.S.
2000 vs 2003	[80,125]	57.5	[28.4,116.5]	N.S.	136.4	[56.1,331.1]	N.S.	102.9	[52.0,203.3]	N.S.	105.3	[50.7,218.5]	N.S.
2000 vs 2004	[80,125]	36.1	[19.4,67.0]	<0.0001	139.2	[64.3,301.2]	N.S.	68.1	[36.8,125.9]	N.S.	103.4	[50.3,212.5]	N.S.
2001 vs 2002	[80,125]	85.8	[43.4,169.6]	N.S.	62.8	[27.0,146.1]	N.S.	48.1	[25.4,91.1]	0.002	100.0	[49.4,202.0]	N.S.
2001 vs 2003	[80,125]	65.7	[32.0,134.7]	N.S.	75.9	[30.8,187.0]	N.S.	70.0	[36.0,135.8]	N.S.	158.4	[76.3,328.7]	N.S.
2001 vs 2004	[80,125]	41.2	[21.9,77.5]	0.000	77.5	[35.2,170.5]	N.S.	46.3	[25.6,83.9]	<0.0001	155.6	[75.7,319.8]	N.S.
2002 vs 2003	[80,125]	76.5	[39.4,148.6]	N.S.	120.8	[53.2,274.6]	N.S.	145.5	[78.0,271.2]	N.S.	158.5	[79.8,314.7]	N.S.
2002 vs 2004	[80,125]	48.0	[27.1,84.8]	<0.0001	123.3	[61.5,247.2]	N.S.	96.3	[55.7,166.8]	N.S.	155.7	[79.2,305.9]	N.S.
2003 vs 2004	[80,125]	62.7	[33.9,115.8]	N.S.	102.1	[47.5,219.2]	N.S.	66.2	[37.2,117.9]	N.S.	98.2	[48.6,198.4]	N.S.

### Comparison of the results obtained with the different methods used

GLSMRs and ANOVA generated consistent results. With the GLM, results were similar to the two other tests for CQ and ART; for MdAQ, the GLM identified only a decrease between 1997 and 2001; for QN, it showed an increase between 1997 and 2001, while the ANOVA and GLSMRs revealed a decrease for 2001–2002 and 2001–2004 periods respectively.

For comparison, when this set of data is analysed with GLSMRs with no random intercept and 95%CIs calculated without adjustment for multiple comparisons, results are slightly different from the current GLSMRs. With the former, significant differences are found for 1997–2000 for CQ and ART, between 2001–2002 for CQ and 2002–2003 for ART. There are two significant differences with 2000 as reference year in comparison with 2002 and 2004, which are not found with the current GLSMRs. Similarly, 2001 is different from 2003 and 2003 from 2004. In the case of ART, the year 2003 is significantly different from the others while it was not with the current GLSMRs

## Discussion

In this study, three different statistical methods to assess changes of IC_50 _over time (ANOVA, GLM, GLSMRs) were compared. The use of data from a single site of moderate to high transmission (25 infecting bites per person-year), and with consistent treatment policies and practices, meant that all patients were expected to be infected with parasites having been under the same degree of drug pressure.

### Normality assumption

Data were not normally distributed, even after log_e_-transformation, for all drugs except log_e_-transformed ART. The ANOVA can be used on non-normally distributed values (although in this case conclusions are less robust), and allows for multiple pair wise comparisons adjustment (but then residuals must be checked for independence and normality as well as homosedasticity, i.e the condition whereby variances are equal.) The GLM requires normally distributed values when using an identity link function (i.e. an assumption of normal distribution for the studied parameter) for comparison with a unique reference (baseline) value. For non-normally distributed data, a different model must be used for each different reference value of the independent factor. The GLSMRs approach can use non normally distributed values and allows adjusted multiple comparisons between years.

### GLSMR

The GLSMR had broader applicability than the other methods because, even if a mixed model is used to obtain the LSMs, it does not require either normally distributed IC_50_s or variance homogeneity. However, one needs to verify the assumptions needed for a mixed model such as the normality of the residuals, the normality of the random effects, and the independence of the residuals and the random effects. As log_e_-transformation serves the purpose of deriving GLSMs of the results, the linear mixed model allowed to relax the assumption of independence of the model residuals and to account for the inherent variability of the data structure in a more flexible way. The *in vitro *test reflects the susceptibility of the whole parasite population in one isolate, and thus cannot separate the effects of the various parasite clones in a given infected subject. Therefore one cannot parameterize the within-subject effect or within-parasite population effect in the linear mixed model. However, it can be argued that these effects are taken into account in the overall mean, which was defined as random in the model.

GLSMRs calculated at a 5% level without an adjustment for multiple comparisons are more likely to wrongly detect a significant difference because of the multiplicity of the statistical tests performed than the GLSMRs at a 1% level and with an adjustment for multiplicity.

### Linear models

ANOVA and pair wise means comparisons between years were good indicators of a difference between years. Noteworthy, in bioequivalence studies GLSMR are generally computed using an ANOVA model, but effects specified as random in a linear model are treated as a fixed factor as they serve the sole purpose of producing the corresponding expected mean squares [20]. These expected mean squares lead to the traditional ANOVA components without accounting for the random effect in the variance.

In the GLSMRs calculations, the mixed linear model was computed using restricted maximum likelihood to evaluate variance parameters, which are in general preferred to ANOVA estimates [20]. Furthermore, mixed models are commonly used when there are different levels of clustering in the observations. The sole level of grouping was the year (treated as fixed in the model), so no other particular variable (or grouping level) was defined as random. The reasons for treating the time variable as discrete (i.e. by calendar year and not as a continuous variable) are: (i) there was a 3-year gap between 1997 (the baseline) and 2000 (the first of a series of five consecutive years.); (ii) only qualitative or discrete variables allowed for the model to simply extract estimates for the different categories of the year effect; (iii) the majority of malaria cases and treatments cluster between July–November during the wet season. Hence, subjects were grouped by year and it was assumed that there was a random part of measurements within years due to the contribution of the subject and the parasite strains subjects were infected with. Specifically, the number of isolates for each year was not the same and IC_50_s varied considerably from year to year (Table 1).

The GLM appears not to be suitable to compare IC_50_s because it treated values as if there were repeat measures from the same subjects, while isolates came from different individuals. In addition, IC_50_s were non-normally distributed despite log_e_-transformation.

### Estimates of the IC50

It is clear that customary approaches are not satisfactory as the difficulty in the analyses is that the data are not time series or longitudinal data. They are also not normally distributed – a necessary condition to use parametric statistical tests, and a critical point of this work. In a recently published paper, Kaddouri *et al *[21] developed a new inhibitory sigmoid Emax statistical model to estimate more precisely the IC_50 _of a given subject. However, it requires cut-off values for resistance of the studied parasites strains to a range of treatment, an element which is not easy to derive for antimalarial drugs. A Bayesian approach was also proposed recently to provide a correction of the estimate of the true IC_50 _[22]. This work was based on the assumption that resistance is systematically overestimated because: (i) the precision of the estimated IC_50 _value of the most resistant isolate will usually be the poorest of all the isolates assayed, (ii) sigmoid curve fitting or probit analysis of a unique isolate takes no account of other isolates in the series tested. This approach requires the distribution of measured IC_50 _to be established before using it to produce a large number of points and estimate the median as the true IC_50_. In doing so, one is inevitably faced with the problem of normality and the choice to apply data transformations such as log or Box-Cox [23]. This work offers an alternative way to deal with data transformation and normality condition in the context of the evolution of microbial susceptibility to drugs.

### Expression of results

GLSMRs were found to be more intuitive, as results are expressed as percentage difference (increase or decrease) between two years, while with LSMs comparisons increases and decreases from the reference appear as inverted (they are marked with a negative and a positive sign, respectively). As geometric means are generally used to express IC_50_s of a pool of isolates, GLSMR are naturally easier to understand and interpret than the other statistical methods tested here.

Two different plots were also produced in order to illustrate the difficulties in interpreting trends over time in drug susceptibility. Figure 1 is a traditional way of plotting the distribution of IC_50_s with cut-off values above which a strain is resistant to a given drug. This makes the reader falsely interpret means against the cut-off, while there is a great variability in the data and no validated thresholds for the majority of antimalarial drugs. In addition, this display does not provide any indication of the significance (or lack thereof) of change between years. Figure 2 is based on the GLSMR results and provides a direct comparison of GLSM between years; it does not need the cut-off values; it depicts visually a statistical test as the confidence intervals of each GLSMR against the line of identity between sets of data (here: years).

## Conclusion

Treatment policy decisions would benefit from reliable information on changes in susceptibility of parasite or bacterial isolates to drugs over time. This entails an adequate statistical method, which can also account for the inherent variability of in vitro drug susceptibility tests (Figure 2). This is particularly important for antimalarial drugs and cases alike where validated thresholds for resistance are not available. The underlying linear mixed model of GLSMRs allowed accounting for this variability and for unequal number of isolates collected during field testing. Based on these data GLSMRs appear to be more accurate and to offer advantages over other tests for the "longitudinal" analysis of IC_50_s. We used a simple statistical model which produces easily interpretable results and can be found in any statistical software. Finally, the utility of GLSMRs in monitoring drug susceptibility of not only malaria parasites but also other microbes should be further tested.

## Competing interests

The author(s) declare that they have no competing interests.

## Authors' contributions

All authors read and approved the final manuscript.

MV conceived the methodology and conducted the analyses; PO contributed to the concept; both contributed to the writing of the paper. PO is a staff member of the World Health Organization. The authors alone are responsible for the views expressed in this publication and they do not necessarily represent the decisions, policy or views of the World Health Organization.
